# Pandemic Influenza Virus Surveillance, Izu-Oshima Island, Japan

**DOI:** 10.3201/eid1811.111681

**Published:** 2012-11

**Authors:** Tomoko Inamasu, Kouji Sudo, Shingo Kato, Hiroshi Deguchi, Manabu Ichikawa, Tadanori Shimizu, Tadami Maeda, Shuhei Fujimoto, Toru Takebayashi, Tomoya Saito

**Affiliations:** Author affiliations: Keio University Global Security Research Institute, Tokyo, Japan (T. Inamasu, K. Sudo, S. Kato, H. Deguchi, T. Saito);; Keio University School of Medicine, Tokyo (K. Sudo, S. Kato, T. Takebayashi, T. Saito);; Tokyo Institute of Technology, Kanagawa, Japan (H. Deguchi, M. Ichikawa);; Oshima Medical Center, Tokyo (T. Shimizu);; Maeda Internal Medicine Clinic, Tokyo (T. Maeda);; Tokai University School of Medicine, Kanagawa (S. Fujimoto)

**Keywords:** Influenza, pandemic, surveillance, A(H1N1)pdm09, Izu-Oshima, island, Japan, viruses

## Abstract

A population-based influenza surveillance study (using PCR virus subtyping) on Izu-Oshima Island, Japan, found that the cumulative incidence of influenza A(H1N1)pdm09 virus infections 2 seasons after the pandemic was highest for those 10–14 years of age (43.1%). No postpandemic A(H1N1)pdm09 case-patients had been infected with A(H1N1)pdm09 virus during the pandemic season.

The dynamics of an influenza epidemic are difficult to determine because they vary for each circulating influenza subtype. We evaluated the epidemiology of influenza A subtypes at Oshima Medical Center and Maeda Internal Medicine Clinic on Izu-Oshima Island, Japan. The island is a semiclosed community, which facilitates population-based surveillance. Access to the island is limited; ≈18,000 persons travel to and from the island each month, primarily to and from Tokyo (1). Izu-Oshima Island is 120 km southwest of Tokyo; population was 8,856 in January 2009.

## The Study

Clinical information was collected retrospectively for January 1 through July 31, 2009, and prospectively from August 1, 2009 through April 30, 2011. Retrospectively, we identified patients who had been tested for influenza by use of a rapid test kit and extracted personal and clinical information from medical records. Prospectively, we collected the same information from patients tested for influenza by rapid test kit and used nasopharyngeal swab extracts from rapid test kits for virus typing. Typing was performed by reverse transcription nested-PCR (RT-nPCR): multiplex for seasonal influenza virus (subtypes A/H1, A/H3, B-, and A/H5) and simplex for influenza A(H1N1)pdm09 virus (Technical Appendix Table 1). By amplifying product exclusively for influenza A(H1N1)pdm09 and for traditional influenza (H1N1) viruses and by producing a different length product for each subtype, this method enabled us to easily define the subtypes visually. We started using RT-nPCR in week 33 of 2009. Protocols were approved by Keio University School of Medicine Ethical Committee.

**Table 1:** Rapid diagnostic test results and prescription of anti-influenza agents in Izu-Oshima

Influenza season and influenza virus type*	Prescriptions written at clinics, no. (%)					Total
	Oseltamivir†	Zanamivir‡	Peramivir§	Laninamivir¶#	None	
2008–09						
A	193 (53.5) §	45 (12.5)§	0	0	124 (34.3)	361
B	39 (32.5)	29 (24.2)	0	0	52 (43.3)	120
A + B	2 (3.3)	0	0	0	4 (66.7)	6
Negative	32 (5.5)	10 (1.7)	0	0	537 (92.8)	579
2009–10						
A	219 (48.5)	209 (46.2)	0	0	24 (5.3)	452
Negative	42 (4.7)	33 (3.7)	0	0	828 (91.7)	903
2010–11						
A	147 (66.2)	52 (23.4)	3 (1.4)	0	20 (9.0)	222
B	60 (38.0)	82 (51.9)	1 (0.6)	1 (0.6)	14 (8.9)	158
Negative	44 (7.2)	18 (3.0)	0	0	548 (89.8)	610

*Influenza virus type determined by rapid diagnostic test. †Tamiflu, Chugai Pharmaceutical Co., Tokyo, Japan. ‡Relenza, GlaxoSmithKline, Research Triangle Park, NC, USA. §Rapiacta, BioCryst Pharmaceuticals Inc., Durham, NC, USA; marketed since January 27, 2010. ¶Inavir, Daiichi Sankyo Co., Ltd., Tokyo, Japan; marketed since October 19, 2010. #IIncludes 1 case for which Tamiflu and Relenza were prescribed.

For the retrospective period, we identified 1,066 suspected cases of influenza; 20 patients were nonresidents of Izu-Oshima Island; the address for 1 patient was unknown. For the prospective period, we identified 2,348 patients with suspected influenza; 3 were excluded because they did not consent to study participation. Patients from the prospective period were tested with a rapid test. In total, 97.8% (2,293/2,345) of the samples were also tested by RT-nPCR. The total number of patients with suspected influenza in the prospective study was 2,219 (a patient with multiple visits within 7 days was counted as 1 patient), of which 78 were not residents of Izu-Oshima Island (Technical Appendix Table 2).

**Table 2:** Incidence of major influenza virus subtypes endemic to Izu-Oshima Island, Japan, by influenza season

Age, y*	Incidence of influenza cases among Izu-Oshima residents, no. (%)											
	2008–09				2009–10			2010–11				Cumulative incidence of A/H1¶
	No.†	Influenza virus type			No.†	Influenza virus type A/H1§		No.†	Influenza virus type			
		A‡	B						A/H1§	A/H3	B	
0–4	326	65 (19.9)	11 (3.4)		323	36 (11.1)		316	20 (6.3)	21 (6.6)	16 (5.1)	41 (13.0)
5–9	366	98 (26.8)	51 (13.9)		367	105 (28.6)		349	39 (11.2)	6 (1.7)	91 (26.1)	137 (39.3)
10–14	316	53 (16.8)	44 (13.9)		316	97 (30.7)		332	26 (7.8)	3 (0.9)	48 (14.5)	143 (43.1)
15–19	475	10 (2.1)	4 (0.8)		480	91 (19.0)		475	24 (5.1)	0	6 (1.3)	129 (27.2)
20–29	541	19 (3.5)	3 (0.6)		492	25 (5.1)		486	12 (2.5)	2 (0.4)	2 (0.4)	39 (8.0)
30–39	987	56 (5.7)	1 (0.1)		991	39 (3.9)		951	22 (2.3)	9 (0.9)	5 (0.5)	62 (6.5)
40–49	977	27 (2.8)	2 (0.2)		971	21 (2.2)		996	18 (1.8)	7 (0.7)	5 (0.5)	42 (4.2)
50–59	1,396	15 (1.1)	1 (0.1)		1,283	14 (1.1)		1,179	10 (1.8)	5 (4.2)	4 (0.3)	24 (2.0)
>60	3,472	12 (0.3)	3 (0.1)		3,540	8 (0.2)		3,568	2 (0.1)	5 (0.1)	2 (0.1)	11 (0.3)
Total	8,856	355 (4.0)	120 (1.4)		8,763	436 (5.0)		8,652	173 (2.0)	58 (0.7)	179 (2.1)	628 (7.3)

*Age during January 2009 (for 2008–09), 2010 (for 2009–10), and 2011 (for 2010–11). †Population recorded in the Resident Registry in January 2009 (for 2008–09), 2010 (for 2009–10), and 2011 (for 2010–11). ‡Influenza A (unspecified). The number of second diagnoses in the season was excluded. §Influenza A(H1N1)pdm09. ¶The sum of influenza A(H1N1)pdm09 cases among residents in Izu-Oshima during the study divided by the population during January 2011.

The sensitivity of the rapid test kit compared with RT-nPCR was ≈90% for type A but lower (≈80%) for type B (Technical Appendix Table 3). Among patients with positive rapid test results, >90% received anti-influenza agents (Table 1). No cases were severe or fatal.

The same influenza subtypes circulated in Izu-Oshima as in other areas (2,3). We assessed the period from when a novel virus was introduced through the postpandemic season. The introduction of A(H1N1)pdm09 virus in Izu-Oshima occurred 11 weeks after confirmation of the first case in Japan (4) and 10 weeks after confirmation in Tokyo (5). After the first case of A(H1N1)pdm09 infection was identified in Japan on May 16 (week 20) (4), no other influenza A cases were diagnosed by a rapid test in Izu-Oshima until August 1 (week 31), when a patient with influenza A (unspecified) was determined to have had contact with a person with confirmed A(H1N1)pdm09 infection on mainland Japan.

**Figure 1:**
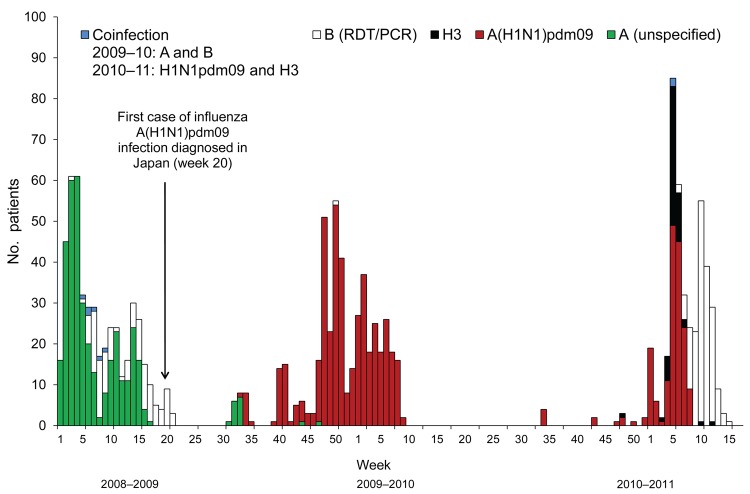
Cases of influenza and influenza-like illnesses on Izu-Oshima Island, Japan, from week 1 of 2009 through week 17 of 2011. The number of influenza cases and influenza-like illnesses are plotted weekly from the disease onset. Influenza cases were defined as illnesses diagnosed by a rapid test combined with a reverse transcription nested PCR (RT-nPCR) or by a rapid diagnostic test (RDT) alone, during the retrospective period (unspecified). Influenza-like illnesses were defined as cases for which influenza was ruled out by negative RT-nPCR or cases for which influenza was ruled out by RDT results and further tests were not performed. Multiple visits within 7 days were counted as a single case.　Disease onset was defined by the date when the patient first reported fever or upper respiratory symptoms. The disease onset for the case that had no date in the clinical records was defined as the day before the first clinical visit according to the median day of visit from the available study data. A and B, co-infection, cases diagnosed by RDT. B (RDT/PCR), cases diagnosed by a RDT or RT-nPCR. A(H1N1)pdm09 and H3, co-infection cases with 2 virus subtypes confirmed by RT-nPCR. Influenza seasons were defined as follows: week 1–30 of 2009 was the 2008–09 prepandemic season, week 31 of 2009–week 33 of 2010 was the 2009–10 pandemic season, and week 34 of 2010–week 17 of 2011 was the 2010–11 postpandemic season.

The first outbreaks of influenza A in Izu-Oshima ceased within 5 weeks (Figure 1). After 3 weeks with no cases, starting at the end of September (week 39), clusters of influenza cases were observed in schools and families. Although immediate school or class closures were implemented, the pandemic began in the middle of November and peaked during week 50, which was 6 weeks later than the Tokyo peak (5). On the island, the incidence of A(H1N1)pdm09 infection during the pandemic season was higher than the incidence caused by other subtypes during the 2009–2011 seasons (Table 2). However, the overall incidence on the island (5.0%) during the pandemic season was one third of the estimated incidence for all of Japan (16.2%) (3).

**Figure 2:**
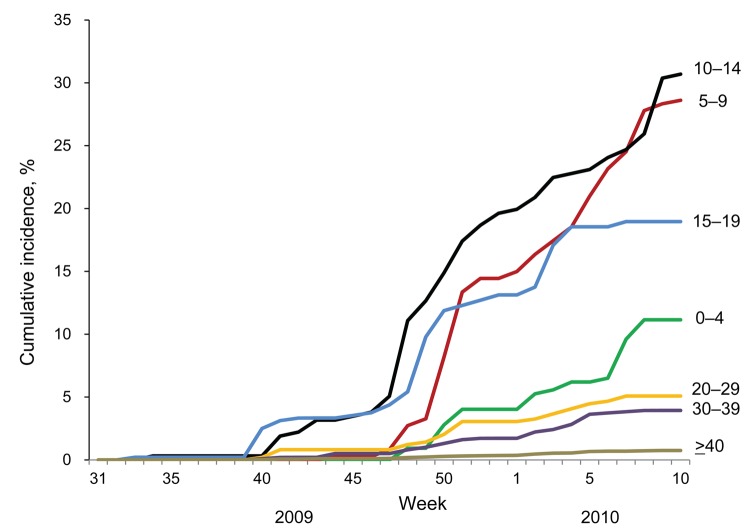
Cumulative incidence of influenza A(H1N1)pdm09 infections by age group during the 2009–10 season. The cumulative incidence of A(H1N1)pdm09 infections for 2009–10 was calculated for the sum of A(H1N1)pdm09 virus cases among residents on Izu-Oshima Island, Japan, divided by the population at the end of December 2009 and plotted by week in the 2009–10 season. The numbers adjacent to the lines indicate the age groups, in years.

The introduction and dissemination of A(H1N1)pdm09 virus varied by age (Figure 2). On the island, as on the mainland, introduction of the emerging virus preceded the outbreak among high school–age children (4,6). However, at the end of the season, the incidence among persons 5–14 years of age exceeded that among persons 15–19 years of age (Table 2).

During the postpandemic season, in addition to A(H1N1)pdm09 virus, epidemics of influenza A/H3 and B viruses occurred. No patient with confirmed A(H1N1)pdm09 infection during the postpandemic season had a history of influenza in the previous season; but 28% (50/180) of patients with influenza B and 3% (2/58) with influenza A/H3 virus did. The cumulative incidence of A(H1N1)pdm09 infection 2 seasons after the pandemic was estimated at 7.3% and was highest (43.1%) among persons 10–14 years of age.

## Conclusions

This population-based surveillance study determined the incidence of the influenza virus subtypes circulating in pandemic and postpandemic seasons. The estimated incidence of symptomatic cases was accurate because of the easy access to health care on Izu-Oshima Island. Care was sought for almost all (97%) children with upper respiratory symptoms and fever, although the proportion of adults who sought clinical care was not high (7). Most (83.6%; 1,774/2,122) patients for whom disease onset was identified had visited a medical institute within 2 days of disease onset despite their symptoms being only mild to moderate. Furthermore, all suspected cases of influenza, except 2, were confirmed by a rapid test; most were tested further and isolates were subtyped by RT-nPCR.

The cumulative incidence 2 seasons after the pandemic indicates that early introduction of A(H1N1)pdm09 virus to those 15–19 years of age was not caused by differential sensitivity to the virus. Rather, it was probably caused by more frequent exposure to the emerging virus, possibly because of higher mobility of persons in this age group. Considering the conservative antigenic property of A(H1N1)pdm09 virus in the postpandemic season (8), the absence of A(H1N1)pdm09 infection in this season among those who had experienced it in the pandemic season suggests that immune memory persisted in the postpandemic season. The cumulative incidence suggests that nearly half of the school-age children had immunity to A(H1N1)pdm09 virus by infection after 2 seasons. The remaining virus-naive elderly population should be considered for future preventive intervention, although they might have some immunity against A(H1N1)pdm09 virus (9–12).

The delayed introduction of A(H1N1)pdm09 virus might primarily be explained by the isolated environment of the island; introduction would be mediated solely by visitors carrying the virus. The delayed start and peak of the epidemic and the low incidence could be attributed early case identification plus early and extensive therapy (including prompt initiation of antiviral medication according to results of proactively performed rapid tests); easy access to health care; and public health interventions (such as school closures).

In addition, unique social features might also have contributed to the delayed pandemic and low disease incidence. The proportion of children <15 years of age (≈12%) is the same on Izu-Oshima Island as in Tokyo; whereas, the proportion of those ≥65 years of age is 31% on the island and only 20% in Tokyo. Assuming that persons ≥65 years of age had preexisting immunity against A(H1N1)pdm09 virus, as suggested by other studies (9–12), the community possibly had a larger number of nonsusceptible persons. Limited public transport and low population density (96/km^2^) might have reduced disease spread. School closures might have more effectively reduced the chance of transmission in such settings than in other areas. This study provides a sound basis for modeling studies that consider social structures to help explain the effects of public health interventions for influenza spread in a community.

Technical Appendix Reverse transcription nested PCR (RT-nPCR) primers used for seasonal (multiplex) and pandemic influenza (simplex) detection and typing (Table 1). Diagnosis of influenza cases and influenza-like illnesses from 2008–09 to 2010–2011 influenza seasons (Table 2). Sensitivity and specificity of QuickNavi-Flu kit (Nordic Biolabs AB, Taby, Sweden) compared with RT-nPCR (Table 3).
