# Supplementary material for: Pandemic Influenza Virus Surveillance, Izu-Oshima Island, Japan
Source: Emerg Infect Dis. 2012 Nov;18(11):1882–5. doi: 10.3201/eid1811.111681 (PMC3559151; doi:10.3201/eid1811.111681)
Supplement: Technical Appendix — Reverse transcription nested PCR (RT-nPCR) primers used for seasonal (multiplex) and pandemic influenza (simplex) detection and typing (Table 1). Diagnosis of influenza cases and influenza-like illnesses from 2008–09 to 2010–2011 influenza seasons (Table 2). Sensitivity and specificity of QuickNavi-Flu kit (Nordic Biolabs AB, Taby, Sweden) compared with RT-nPCR (Table 3). [file 11-1681-Techapp-s1.pdf]

Article DOI: http://dx.doi.org/10.3201/eid1811.111681

Pandemic Influenza Virus Surveillance, Izu-Oshima Island, Japan

Technical Appendix

Table 1. Reverse transcription nested PCR primers used for seasonal (multiplex) and pandemic influenza (simplex) detection and typing

Seasonal flu primers (RT-1st)*	Sequence, 5′→3′
Flu_A_H1/N1_1F	GTR TCR GCA TCA TGC TCC CAT AA
Flu_A_H1/N1_1R	GAT TCC TGA YCC AAA GCC TCT AC
FluA H3 1F	TTT GTT GAA CGC AGC AAA GC
FluA H3 1R	TGT CTC CCG GTT TTA CTA TTG TCC
FluB 1F	AAA DGC ACC AGG AGG ACC CT
FluB 1R	TCT GAA TGG AAC CCC CAA AC
FluA H5 1F	AAG YTA CAA TAA TAC CAA CCA AGA AGA TCT
FluA H5 1R	AGT TGA CCT TAT TGG TGA CTC CAT C
Seasonal flu primers (nested) †	
Flu_A_H1/N1_2F	ARA AAT TTG CTA TGG CTG ACG G
Flu_A_H1/N1_2R	ATC CCC GGG TTC MAG CAG A
FluA H3 2F	CGG ATT ATG CCT CCC TTA GGT C
FluA H3 2R	CCT GGG TCT AGA YCC GAT ATT CG
FluB 2F	CAG ACT TGG AAC CTC AGG RTC TTG
FluB 2R	CCC CTT CTG TAC AAA TGT RTG GTA CTT C
FluA H5 2F	TTC CRT TGG GAC ATC AAC ACT
FluA H5 2R	TCT ACC ATT CCC TGC CAT CC
A/H1N1(2009) primers (RT-1st)* ‡	
Flu_A_H1/N1_2009_1F	GTA ACG GCA GCA TGT CCT CAT GC
Flu_A_H1/N1_2009_1R	AAT ACC AGA TCC AGC ATT TCT TT
A/H1N1(2009) primers (nested) †‡	
Flu_A_H1/N1_2009_2F	AAA AAT TTA ATA TGG CTA GTT A
Flu_A_H1/N1_2009_2R	GTC TCC CGG CTC TAC TAG T

*Thermal cycling for the 1st cycle was 50°C for 10 min (for the reverse transcription), 94°C for 2 min (for degeneration), 94°C for 5 s, 48°C for 10 s, 72°C for 15 s x 5 cycles, 94°C for 5 s, 60°C for 10 s, 72°C for 15 s x 25 cycles, and 72°C for 1 min.

†Thermal cycling for the nested cycle was 94°C for 2 min (for degeneration), 94°C for 5 s, 48°C for 10 s, 72°C for 15 s x 5 cycles, 94°C for 5 s, 60°C for 10 s, 72°C for 15 s x 25 cycles, and 72°C for 1 min.

‡The specificity and sensitivity of the reverse transcription nested-PCR (RT-nPCR) method was determined by comparing the respective test results for 337 samples from Keio University Hospital, Tokyo, Japan, (during the pandemic season) with those obtained using the Real Time Ready Swine Inf A/H1N1 Detection Set (Roche Kit) (Roche) (*1*). The agreement between the RT-nPCR and the Roche Kit for A(H1N1)pdm09 was 98%. From 266 samples that had been typed negative by the Roche Kit, eight samples were shown to be positive for A(H1N1)pdm09 using RT-nPCR. Direct sequencing of 3 out of 8 samples that had discrepant results showed that the 3 samples were true A(H1N1)pdm09 positives. Therefore, the RT-nPCR method has a higher detection sensitivity for A(H1N1)pdm09 than the Roche Kit.

Table 2. Diagnosis of influenza cases and influenza-like illnesses from 2008/2009 to 2010/2011 seasons

	No. cases by subtype and by flu season (%)		
Diagnosis	2008/2009* (pre-pandemic)	2009/2010† (pandemic)	2010/2011‡ (post-pandemic)
Influenza	487	467	416
Diagnosis by Rapid Diagnostic Test§			
A (unspecified)	361 (74.1)	16 (3.4)	0
B	120 (24.6)	0	1 (0.2)
A&B	6 (1.2)	0	0
Diagnosis by RT-nPCR			
A/H1N1 seasonal	–	0	0
A(H1N1)pdm09	–	450 (96.2)	176 (42.3)
A/H3	–	0	58 (13.9)
A(H1N1)pdm09 and A/H3	–	0	2 (0.5)
A/H5	–	0	0
B	–7	1 (0.2)	179 (43)
Influenza-like illness¶	579	803	533
Total	1,066	1,270	949

*2008/2009: week 1 in 2009 to week 30 in 2009

†2009/2010: week 31 in 2009 to week 33 in 2010

‡2010/2011: week 34 in 2011 to week 17 in 2011

§Cases for which PCR was not performed.

¶Influenza-like illness is defined as cases where influenza was ruled out by negative RT-nPCR results or cases where influenza was ruled out using rapid tests and where further tests were not performed.

Table 3. Sensitivity and specificity of QuickNavi-Flu kit compared with RT-nPCR

Season	Virustype	Sensitivity, % (95%CI)*	Specificity, % (95% CI)*
2009/2010	Any Type A	89.6 (86.5–92.2)	99.1 (98.2–99.7)
(n = 1,300)†	A(H1N1)pdm09	89.6 (86.5–92.2)	–
2010/2011	Any Type A	92.1 (87.9–95.1)	99.7 (99.0–100)
(n = 982)†	A(H1N1)pdm09	92.2 (87.2–95.7)	–
	A/H3	91.9 (82.2–97.3)	–
	Type B	80.1 (73.8– 85.5)	100 (NA)

*The sensitivity and specificity of the rapid test QuickNavi-Flu kit (DENKA SEIKEN Co., Ltd, Tokyo, Japan) was calculated in comparison to RT-nPCR results that were used as the standard.

†A QuickNavi rapid diagnostic kit was used for 99.4% (2,334/2,345) of samples obtained from clinical visits where influenza was suspected in the 2009–2011 seasons: 97.8% (2,282/2,334) of samples were tested further by RT-nPCR. Eleven other samples were tested by Clearview Exact Influenza A/B (Inverness Medical, Co., Ltd, Japan); these results matched the RT-nPCR results except for one false negative.

Reference

1. Wenzel JJ, Panning M, Kaul KL, Mangold KA, Revell PA, Luna RA, et al. Analytical performance determination and clinical validation of the novel Roche RealTime Ready Influenza A/H1N1 Detection Set. J Clin Microbiol. 2010;48:3088–94. PubMed http://dx.doi.org/10.1128/JCM.00785-10
